# On the Physical Education and Training of Children

**Published:** 1873-01

**Authors:** H. W. Boone

**Affiliations:** San Francisco


*On the Physical Education and Training of Children.* By H. W. Boone, M.D., San Francisco.

A very suggestive writer remarks, the first requisite of success in life is "to be a good animal;" and to be a nation of good animals is the first condition of national prosperity. The event of war often turns on the strength and hardiness of soldiers; and the contests of commerce are greatly decided by the bodily endurance of producers. Under the keen competition of life few of us can stand the application required without more or less injury. Thousands break down under the strain they are subjected to. This makes it specially important that children should be trained with a view to fit them physically, as well as mentally, to bear the wear and tear of the life they will have to encounter. Fortunately this matter is attracting much more attention than it did formerly, and we propose some statement of the views sanctioned by those who take the lead in the discussion of this subject.

The great attention which has been paid to the improvement of stock, has shown how all the qualities of strength, speed and endurance can surely be enhanced by attention to proper laws, and that a nobler and finer animal may be developed. The same rule applies to man, for it is a fact which no one will dispute, that man is subject to the same organic laws as the lower creatures.

First let us consider the subject of nutrition. While in a former age there was a tendency to general over-eating, there seems now to be a reaction, and more particularly among the educated classes it is to be feared that children do not get either the quantity or quality of food best suited to the wants of their growing organisms. While over-feeding and under-feeding are both bad, the latter is far the worst. The natural appetites are a very good guide, and it is only where they have been pampered that they fail to give proper indications. The child who has been fed on a liberal supply of wholesome, nutritious food, and always has enough, is not in danger of over-eating itself, while, if not treated to dainties, it will not clamor for things it has no idea of. Some of the things universally desired by children are sternly denied them by most parents, from mistaken notions and the force of traditions. All children are fond of sweets, and physiology teaches us what an important part sugar plays in the vital processes; together with fat, it is eventually oxydized, and there is an accompanying evolution of heat. The researches of Claude Bernard go to show that sugar is produced in very great quantity by the liver, and must have some important function to perform. When we see children with a strong desire for this kind of food, and note at the same time that they have usually a marked dislike to that food which gives out the greatest amount of heat during its oxidation, namely, fat, there is reason to think that excess of one compensates for defect of the other; that the organism demands more sugar, because it cannot deal with so much fat.

Then children are fond of vegetable acids. Vegetable acids are good tonics, and ripe fruit aids digestion, and is especially valuable in regulating the bowels. There is no reason why children should not partake of both sweets and vegetable acids. On the contrary, within due bounds, the assimilation of both is advantageous and even necessary. And yet there is a general tendency to forbid their use to children, who consequently eat green fruit, or gorge themselves with sweets whenever they get a chance, and the illness following unwonted excesses is made the justification for a total exclusion of these articles from their dietary. With equal justice might it be argued that, as unaccustomed exertion produces depression and fatigue, all exercise is pernicious, and should be prohibited. Neither parents nor physicians can get up an arbitrary standard of feeding, and expect that all children will be benefited by it. The demand of the system for food varies with the temperature, the hygrometric condition of the air, with the electric state of the air; varies according to the exercise taken, the kind and quality of food eaten at the last meal, and the rapidity with which that meal was digested. How can we judge of all these things? Nature has provided every little mortal with the same means of sensation as we have, and our function should be neither to check nor force appetite, but to supply the proper and requisite nourishment. The opinion is often held, that children should have little animal food; "meat is not good for little boys and girls," is a very common answer to children. While the stomach of a very young child has not the muscular power to triturate this kind of food, the same objection does not tell against the use of it by those who are a little older. The opinion of science is exactly the opposite of the popular opinion on this question. Each day the body of a man undergoes a certain wear and tear—wear through muscular exertion, wear of the nervous system, wear of viscera in carrying on the functions of life, and this waste has to be renewed. Every day, by radiation his body loses heat, and as the temperature must be maintained, this loss must be compensated by the constant production of heat. The man has simply to maintain his equilibrium. The boy also wastes his tissues by action; he loses heat by radiation, and as his body exposes a greater surface in proportion to its mass than does that of an adult, and loses heat faster, he requires proportionally more calorific food than a grown person. Even if the boy had no more vital processes to carry on than the man, he needs, in proportion to his size, more nutriment. But, in addition, the boy has to grow, or in other words, make new tissue. After providing for all the other needs, the surplus nutriment is wanted to build up the frame, and only by this surplus is normal growth possible. Any growth taking place in the absence of such surplus, causes a manifest prostration, consequent upon deficient repair.

Admitting, as we must, this relatively greater need for nutriment, the question is, how shall we meet it, by giving a large quantity of light food, or a smaller amount of concentrated food? Shall we meet the needs of the child by giving a sufficient quantity of food as good as that of adults, or tax the stomach with a larger quantity of inferior nutriment? The more we save from the labor of digestion, the more energy we leave for purposes of growth and action. The function of nutrition cannot proceed without a large supply of blood and nervous power, and the more nutritious and the better adapted the food is for assimilation, the less extra energy will be taken away from the general fund. Not only the size but the energy of a child depend in great measure on its food, and as that is stronger and more nourishing, so will he be more capable of exertion and endurance. The well known fact of the superior powers of the English navvy to perform hard labor, has long been recognized, but, as stated in Flint's Physiology, this has been shown to turn upon difference of diet and not of race. It has been proved by actual experience that continental navvies, fed in the same way as the English ones, are capable of the same amount of labor. The food of the child should contain fresh meat, vegetables and fruit, and the aim of supplying the nutritive needs of the growing economy by a highly nourishing and somewhat concentrated food, so prepared as to be easily digested, must be kept steadily in view.

Parents often make the mistake of allowing too much sameness in the food of their children. The natural repugnance to a long continuance of one kind of food is a normal incentive to that diversity which is a physiological need of the system. There is no one food, no matter how good, which supplies *all* the requirements of nutrition. The enjoyment of a change of diet is a nervous stimulus, which increases the action of the heart, and by propelling the blood with more vigor, assists digestion. This is practically so well known that every intelligent farmer understands the importance of varying the food of his cattle. Not only should there be variety from time to time, but at *each* meal. The experiments of Goss and Stark (Cyc. Anat. and Physiology), "afford the most decisive proof of the advantage, or rather the necessity of a mixture of substances in order to produce the compound which is the best adapted for the action of the stomach." It may perhaps be necessary to remark that children who have been accustomed to too low a diet, or one made up of innutritious substances in large amount, should not be put on a full regimen too suddenly, as they would suffer from the unaccustomed change. Neither should the concentration be carried too far; a mixed diet, vegetable and animal, will best fulfill all indications.

The strange antagonism to the guidance furnished by the sensations of children, again peeps out in the tendency to clothe them too lightly. The common notion of hardening is a melancholy error. The writer has seen death follow the well-meant attempts of parents to carry out this system, and has witnessed the terrible remorse of a father, who is firmly convinced that he was himself the cause of the early death of his only son. Those who survive carry the marks of their suffering either in growth or constitution. When the constitution is sound enough to stand it, exposure produces hardness, but at the expense of growth. The Shetland pony is tough but a dwarf, and the Laplander and Esquimaux are stunted. This is most easily explained. As we stated before, in order to compensate for the cooling by radiation which the body is ever undergoing, there is an oxidation of certain matters forming part of the food. In proportion as the loss of heat is great, must the quantity of these matters for oxidation be increased. All the energies being drawn upon to maintain the bodily temperature less material is left to build up the frame. This shows the true importance of clothing. Liebig says: "Our clothing is, in reference to the temperature of the body, merely an equivalent for a certain amount of food." By lessening the loss of heat, it lessens the amount of fuel needed to keep up that heat; and the stomach can do more in preparing other materials. Here again the farmers have found out that cattle are fattened on less food in a warm stall, than if the temperature is low, and govern themselves accordingly. In proportion to their smallness and the rapidity of their growth, do children suffer the greatest injury from cold. Quetelet has pointed out, "that in Belgium two infants die in January for one that dies in July." We have before stated that the child exposes more surface in proportion to its bulk than the man, and loses relatively more heat. Lehman says: "If the carbonic acid excreted by young children or animals is calculated for an equal bodily weight, it results that children produce nearly twice as much acid as adults." This is a fair indication of the amount of heat produced. And we see that in children the system has naturally to provide nearly double the proportion for generating heat. *Every ounce* of nourishment needlessly wasted to keep up the temperature, is stolen from the nourishment of the frame, and the child, with lowered vitality, is peculiarly liable to attacks of disease, and has not the power to rally from them. Dr. Combe gives us the true principle in the following words: "The rule is, therefore, not to dress in an invariable way in all cases, but to put on clothing in kind and quantity *sufficient, in the individual case, to protect the body effectually from an abiding sensation of cold, however slight.*" Fashion, which dresses children to please the eye, and changes the ordinary woolen dress for a white one to go out visiting, has, with the aid of the bleak winds of our coast, sent many a little one to Heaven; and perhaps, in its eventualities, the fact that many more have survived, with impaired vigor and weakened forces, mental as well as bodily, to transmit in their turn these failings to their children, is still more sad to contemplate. The clothing of children should never be in such excess as to prove oppressive, but it should always be enough to prevent any general feeling of cold; it should be a good non-conductor, as woolen, strong enough to stand hard play, and its colors such as will not suffer from use and exposure.

Most persons are aware of the importance of bodily exercise for children, yet still they are not generally allowed full scope for the exercise of their energies in this direction. Especially in our cities children are kept at home; it is not genteel to play in the streets; and then they will soil their clothes! While boys manage to get a tolerable amount of exercise, our girls are fettered on every side. Girls must not be allowed to run wild; they will be as rude as boys, and grow up romps, say the defenders of maiden propriety. This fear is needless; the sportive activity of boys does not keep them from growing up into gentlemen. Why should it prevent girls from becoming ladies? Youths often show a ludicrous anxiety to avoid whatever is not manly. It is notorious that English girls are less forward, remain children longer than ours do, and they are certainly modest and reserved enough when they grow up; yet they habitually take an amount of exercise such as our girls never dream of, and are allowed to follow their natural inclinations for juvenile sports. The robust form of the young English lady who walks ten miles for pleasure, and does not suffer from exposure to the inclemency of the weather, is a commentary on the wisdom of their early training.

Gymnastics have been brought in to supplement that natural play which all children seek if allowed to. That this is better than nothing, we admit; but that it is an adequate substitute for play, we deny. These formal muscular actions are less varied than those accompanying juvenile sports, do not secure so equal a distribution of action to all parts of the body; this produces fatigue sooner, and leads to disproportionate development. Gymnastics are inferior in the *quantity* and also in the *quality* of muscular exertion which they secure. They are monotonous, while sports produce exhilaration which has a highly invigorating influence. Happiness is the most powerful of tonics; it tends alike to increase health when it exists, and to restore it when it has been lost.

A topic remains, perhaps demanding more consideration than any of the foregoing. Are the younger adults, and those verging upon maturity, among the educated classes, as strong and as well grown as their seniors? As measured by ancient armor, modern men are proved to be larger than ancient men. The tables of mortality show rather an increase in the duration of life; and yet omitting the laboring classes, it will be found that in a majority of cases children do not reach the stature of their parents; and in massiveness, making due allowance for difference of age, there is the same inferiority. In health the contrast is even greater. Men of past generations, living riotously, could bear more than men of the present generation who live soberly. The annals of the bench and bar show that our recent ancestors were capable of prolonged application without injury, even to very old age. Yet paying more attention to the laws of health, we are continually breaking down under our work. What does this mean? Is it that the past over-feeding, of adults and juveniles, was less injurious than the under-feeding we have spoken of? Is deficient clothing to blame? Is discouragement of juvenile sports, in deference to a false refinement, the cause? Each of these probably has had a share in producing the evil; but another detrimental influence is at work, perhaps more potent than any of the others, except mental application.

The pressure of modern life continually increases the strain on old and young. In all professions and business pursuits, intense competition taxes the abilities and energies of every grown person. In order to fit the young to maintain their place under the competition, they are subjected to more severe discipline than before. Fathers, hard pressed by competitors, have to keep up a more expensive style of living; and, always at work, get little exercise, and few holidays. Their constitutions, broken by over application, they transmit to their children, and these comparatively feeble children, predisposed to break down, are required to go over a more extended course of study than their stronger predecessors. The disastrous effects of this cumulative transgression can be foretold, and every one sees the results. Cases of children or youths of either sex, more or less injured by undue study, frequently come under our notice. Here a child has to leave school; again, you find chronic congestion of the brain. One has a fever, or another is subject to fainting fits. These troubles become hereditary, and the children of parents who have been affected by such complaints, inherit an enfeebled brain, and are frequently unable to bear even a moderate amount of study without headache or vertigo. Indigestion, constipation, cold extremities, palpitation, impaired vision, checked growth, and lax tissue, are only a few of the evils entailed. If sufferings so conspicuous are frequent, how general must be lesser injuries. For one case where well marked injuries are traceable to overwork, there are many cases where the evil is unobstrusive and slowly accumulating, cases of derangement of function attributed to anything but the right cause, cases of retardation and arrest of bodily growth, where a tendency to consumption is induced; most common of all perhaps the predisposition to cerebral disease, which strikes down so many among our overworked upper classes.

When we see the frequent ailments of overworked professional and mercantile men, and how constitutions are undermined by these causes, think upon the sad effects which undue application must produce upon the undeveloped systems of children and young persons. The young are not competent to bear as much hardship or physical exertion, nor as much mental exertion as grown persons. If the full grown suffer so manifestly from the mental exertion required of them, what must be the damage which a mental exertion, often equally excessive, inflicts upon the young? Does not every professional man know how common slight curvature of the spine is among girls, and that this is frequently due to debility? Most parents seem to know the bad effects that follow infant precocity, yet it appears not to be perceived that throughout the whole time of growth a forced developing of intelligence will entail evil results. There is a natural order and a natural rate at which the faculties unfold. If these faculties are overtaxed, the result will be some form of evil. Let it never be forgotten how many demands there are upon the system of the child—repair of muscular tissue, repair of brain and nervous tissue, supply of heat, performance of digestion, growth; we can overstimulate the brain only at the expense of all the other functions, for the amount of vital energy which the body possesses is limited, it is impossible to get more than a certain amount of power. Says a distinguished writer, "It is a physiological law, first pointed out by M. Isidore St. Hiliare, and to which attention has been drawn by Mr. Lewes in his essay on *Dwarfs and Giants*, that there is an antagonism between *growth* and *development*. By growth, as used in this antithetical sense, is to be understood *increase of size;* by development, *increase of structure*, and the law is, that great activity in either of these processes involves retardation or arrest of the other." "Now this law is true, not only of the organism as a whole, but of each separate part. The abnormally rapid advance of any part in respect of structure involves premature arrest of its growth; and this happens with the organ of the mind as certainly as with any other organ. The brain, which, during early years, is relatively larger in mass but imperfect in structure, will, if required to perform its functions with undue activity, undergo a structural advance greater than is appropriate to the age; but the ultimate effect will be a falling short of the size and power that would else have been attained." "And this is a part cause—probably the chief cause—why precocious children, and youths who, up to a certain time, were carrying all before them, so often stop short and disappoint the high hopes of their parents." Prolonged exertion of the brain, then, and undue mental excitement in children and youths, will surely entail accumulating degeneracy of physique. The effects must be bad when a weakened stomach supplies the growing body with blood deficient in quantity and poor in quality, while a weakened heart fails to propel this vitiated current at the normal rate. This system is a mistake, no matter how you regard it. If the mind is plied with facts faster than they can be grasped, they fall out of recollection. It makes study distasteful, and causes an aversion to all books and information in after life, thus defeating its own end.

Acquisition of knowledge is not everything; it is the organization of knowledge we want. Spencer remarks, "It is not the knowledge stored up as intellectual fat which is of value; but that which is turned into intellectual muscle." But its worst feature is that it destroys that physical vigor necessary to make intellectual training useful in the competition of life. Success depends more on energy than upon information by itself. Strong will, and that untiring activity which comes from abundant animal vigor, will gain a victory over competitors enfeebled by over study. Even supposing wordly success attained, the ill health accompanying this system will be a perpetual curse.

On women this system produces even worse results than on men. Men care little for learned women, but they do care for physical beauty, sense, and good spirits. Rosy cheeks, laughing eyes, and a fine figure, gentleness, and good breeding, will carry the day over all the refinements of school lore. As Mr. Zulliver says, "An over cute woman is no better nor a long tailed sheep. She'll fetch none the bigger price for that." Wisely and well it is so ordered, let us remember, that nature's chief end is the welfare of posterity, and so far as that is concerned, mere culture accompanied by a bad physique, is worthless, the descendants of such parents will die out in one or two generations. A good physique is worth preserving, even with poor mental endowments, for throughout future generations the mind may be developed. The system which would overload a girl's memory at the expense of her constitution, is fatal. Educate by all means, and as highly as is compatible with the general health and function of growth. Were the cramming system less pursued, and the system of self-education and reasoning more developed, a high grade of culture might be reached. The pupil would have sufficient interest in her studies to prosecute them when the restraints of tuition are removed, there would be something to fill the blank between the school room and married life. But so to educate as to produce physical degeneracy, is to defeat the very end for which all this toil, expense and solicitude are undergone. This high-pressure system applied to girls, often ruins their prospects for life; besides inflicting on them bad health, with its pains and disabilities, and consequent exacerbation of temper, it very often actually consigns them to celibacy.

Dr. Nathan Allen, of Lowell, brings one part of this subject very forcibly before us. He has studied the subject statistically, and has discovered that the average number of children to American families in New England is but 3 or 3½, against an average of 8 to families of a corresponding social scale a century ago. Population can hardly be kept up among the educated classes, unless an average of more than three children are born to a family. This, then, is the great problem of the future. Historians long debated the question, what was the reason for the decline of Greece and Rome. Prof. Seelye, the eloquent author of "Ecce Homo," has answered this question: "Rome died for want of men." The human harvest was bad. Dr. Allen has shown that, in all probability, Greece died from the same cause as Rome. Practitioners in New York and its vicinity state the same facts. The editor of the *N. Y. Medical Record*, very truly says: "A nation can do without high culture; it can do without extensive territory; it can do for a time without religion; it can, like China, do without a pure morality, *but human beings it must have.* Questions of science, of education, of government, of religion, of morality, etc., are trifles compared with this one great question of population. The time must come when physicians will evince a disposition to employ a portion of their leisure in studying the great laws of population."

The general conclusions to be derived from what we have stated are, that the ordinary treatment of children is often injudicious. Their food is deficient in quantity or quality, or both; they are not properly clothed; they do not have sufficient exercise, more particularly the girls, and the mind is overworked at the expense of the body. We tax the young organism too much, and give it too little. Through infancy, childhood and youth, growth is the requirement to which everything else should be subordinate. The mental and physical activities should be increased gradually only as the rate of growth diminishes. Herbert Spencer, to whose writings we are greatly indebted, remarks: "We do not yet sufficiently realize the truth that as, in this life of ours, the physical underlies the mental, the mental must not be developed at the expense of the physical." All neglect of the laws which conserve or promote health is *physical sin.* When the preservation of health is looked upon as one of our most important *duties to ourselves and others*, the physical training of the young will be understood better, and will receive that attention which it imperatively demands.—*Western Lancet.*

